# Supplementary material for: Saccharomyces boulardii (CNCM I-745) improves intestinal damage in sepsis by remodeling intestinal flora structure
Source: Front Cell Infect Microbiol. 2025 Jul 31;15:1602792. doi: 10.3389/fcimb.2025.1602792 (PMC12350329; doi:10.3389/fcimb.2025.1602792)
Supplement: Supplementary file 1 [file DataSheet1.zip › summary/summary/8_Advanced_analyse/2_PICRUSt2_result/METACYC/Group.CW4_vs_CW0/PICRUSt2_METACYC_Group.CW4_vs_CW0_diff.pdf]

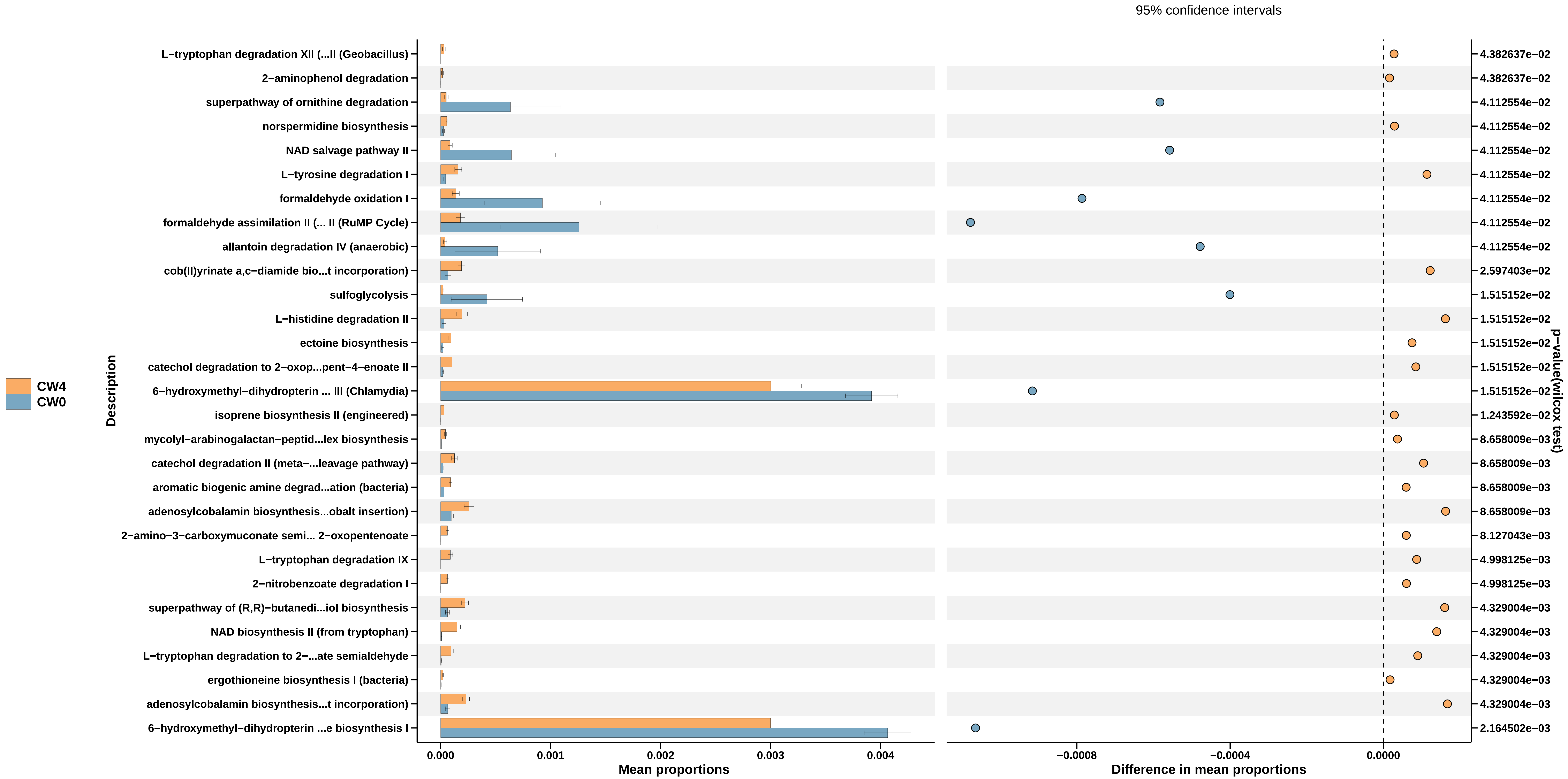

95% confidence intervals
CW4
CW0
Description
L−tryptophan degradation XII (...II (Geobacillus)
2−aminophenol degradation
superpathway of ornithine degradation
norspermidine biosynthesis
NAD salvage pathway II
L−tyrosine degradation I
formaldehyde oxidation I
formaldehyde assimilation II (... II (RuMP Cycle)
allantoin degradation IV (anaerobic)
cob(II)yrinate a,c−diamide bio...t incorporation)
sulfoglycolysis
L−histidine degradation II
ectoine biosynthesis
catechol degradation to 2−oxop...pent−4−enoate II
6−hydroxymethyl−dihydropterin ... III (Chlamydia)
isoprene biosynthesis II (engineered)
mycolyl−arabinogalactan−peptid...lex biosynthesis
catechol degradation II (meta−...leavage pathway)
aromatic biogenic amine degrad...ation (bacteria)
adenosylcobalamin biosynthesis...obalt insertion)
2−amino−3−carboxymuconate semi... 2−oxopentenoate
L−tryptophan degradation IX
2−nitrobenzoate degradation I
superpathway of (R,R)−butanedi...iol biosynthesis
NAD biosynthesis II (from tryptophan)
L−tryptophan degradation to 2−...ate semialdehyde
ergothioneine biosynthesis I (bacteria)
adenosylcobalamin biosynthesis...t incorporation)
6−hydroxymethyl−dihydropterin ...e biosynthesis I
0.000
0.001
0.002
0.003
0.004
Mean proportions
−0.0008
−0.0004
0.0000
Difference in mean proportions
p−value(wilcox test)
4.382637e−02
4.382637e−02
4.112554e−02
4.112554e−02
4.112554e−02
4.112554e−02
4.112554e−02
4.112554e−02
4.112554e−02
2.597403e−02
1.515152e−02
1.515152e−02
1.515152e−02
1.515152e−02
1.515152e−02
1.243592e−02
8.658009e−03
8.658009e−03
8.658009e−03
8.658009e−03
8.127043e−03
4.998125e−03
4.998125e−03
4.329004e−03
4.329004e−03
4.329004e−03
4.329004e−03
4.329004e−03
2.164502e−03
